# Supplementary material for: HMGB1 signaling phosphorylates Ku70 and impairs DNA damage repair in Alzheimer’s disease pathology
Source: Commun Biol. 2021 Oct 11;4:1175. doi: 10.1038/s42003-021-02671-4 (PMC8505418; doi:10.1038/s42003-021-02671-4)
Supplement: Supplementary file 2 — Supplementary Information [file 42003_2021_2671_MOESM2_ESM.pdf]

HMGB1 signaling phosphorylates Ku70 and impairs DNA damage repair in Alzheimer's disease pathology

Hikari Tanaka[1,#], Kanoh Kondo[1,#], Kyota Fujita[1,#], Hidenori Homma[1,#], Kazuhiko Tagawa[1], Xiaocen Jin[1], Meihua Jin[1], Yuki Yoshioka[1], Sumire Takayama[1], Hitomi Masuda[2], Rie Tokuyama[2], Yukoh Nakazaki[2], Takashi Saito[3], Takaomi Saido[4], Shigeo Murayama[5,6], Teikichi Ikura[7], Nobutoshi Ito[7], Yu Yamamori[8], Kentaro Tomii[8], Marco E. Bianchi[9], and Hitoshi Okazawa[1,$]

[1]Department of Neuropathology, Medical Research Institute and Center for Brain Integration Research, Tokyo Medical and Dental University, 1-5-45 Yushima, Bunkyo-ku, Tokyo 113-8510, Japan.

[2]Chiome Bioscience Inc., Sumitomo Fudosan Nishi-shinjuku Bldg. No.6, 3-12-1 Honmachi, Shibuya-ku, Tokyo 151-0071 Japan.

[3]Department of Neurocognitive Science, Institute of Brain Science, Nagoya City University Graduate School of Medical Sciences, 1 Kawasumi, Mizuho-ku, Mizuho-cho, Nagoya, Aichi 467-8601, Japan

[4]Laboratory for Proteolytic Neuroscience, RIKEN Center for Brain Science, 2-1 Hirosawa, Wako, Saitama 351-0198, Japan

[5]Department of Neuropathology, Tokyo Metropolitan Institute of Gerontology, 35-2 Sakae-cho, Itabashi-ku, Tokyo, 173-0015, Japan.

[6]Brain Bank for Neurodevelopmental, Neurological and Psychiatric Disorders, Molecular Research Center for Children's Mental Development, United Graduate School of Child Development, Osaka University, Suita, Osaka, Japan

[7]Department of Structural Biology, Medical Research Institute, Tokyo Medical and Dental University, 1-5-45 Yushima, Bunkyo-ku, Tokyo 113-8510, Japan.

[8]Intelligent Bioinformatics Research Team, Artificial Intelligence Research Center, National Institute of Advanced Industrial Science and Technology, 2-4-7, Aomi, Koto-ku, Tokyo 135-0064, Japan

[9]Division of Genetics and Cell Biology, IRCCS San Raffaele Scientific Institute, Milan, Italy

[#]Contributed equally

[$]Correspondence should be addressed to H.O.

E-mail: okazawa-tky@umin.ac.jp

Ku70 (XRCC6) human & mouse

```
SP|P12956|XRCC6_HUMAN MSGWESYYKTEGDEEAEEEQEENLEASGDYKYSGRDSLIFLVDASKAMFESQSEDELTPF 60
SP|P23475|XRCC6_MOUSE MSEWESYYKTEGEEEEEE--EESPDTGGEYKYSGRDSLIFLVDASRAMFESQGEDELTPF 58
                      ** *********:** **  **. ::.*:****************:******.*******

SP|P12956|XRCC6_HUMAN DMSIQCIQSVYISKIISSDRDLLAVVFYGTEKDKNSVNFKNIYVLQELDNPGAKRILELD 120
SP|P23475|XRCC6_MOUSE DMSIQCIQSVYTSKIISSDRDLLAVVFYGTEKDKNSVNFKNIYVLQDLDNPGAKRVLELD 118
                      *********** ***********************************:********:****

SP|P12956|XRCC6_HUMAN QFKGQQGQKRFQDMMGHGSDYSLSEVLWVCANLFSDVQFKMSHKRIMLFTNEDNPHGNDS 180
SP|P23475|XRCC6_MOUSE QFKGQQGKKHFRDTVGHGSDYSLSEVLWVCANLFSDVQLKMSHKRIMLFTNEDDPHGRDS 178
                      *******:*:*:* :***********************:**************:***.**

SP|P12956|XRCC6_HUMAN AKASRARTKAGDLRDTGIFLDLMHLKKPGGFDISLFYRDIISIAEDEDLRVHFEESSKLE 240
SP|P23475|XRCC6_MOUSE AKASRARTKASDLRDTGIFLDLMHLKKPGGFDVSVFYRDIITTAEDEDLGVHFEESSKLE 238
                      **********.*********************:*:******: ****** **********

SP|P12956|XRCC6_HUMAN DLLRKVRAKETRKRALSRLKLKLNKDIVISVGIYNLVQKALKPPPIKLYRETNEPVKTKT 300
SP|P23475|XRCC6_MOUSE DLLRKVRAKETKKRVLSRLKFKLGEDVVLMVGIYNLVQKANKPFPVRLYRETNEPVKTKT 298
                      ***********:**.*****:**.:*:*: ********** ** *::*************

SP|P12956|XRCC6_HUMAN RTFNTSTGGLLLPSDTKRSQIYGSRQIILEKEETEELKRFDDPGLMLMGFKPLVLLKKHH 360
SP|P23475|XRCC6_MOUSE RTFNVNTGSLLLPSDTKRSLTYGTRQIVLEKEETEELKRFDEPGLILMGFKPTVMLKKQH 358
                      ****..**.**********  **:***:*************:***:****** *:***:*

SP|P12956|XRCC6_HUMAN YLRPSLFVYPEESLVIGSSTLFSALLIKCLEKEVAALCRYTPRRNIPPYFVALVPQEEEL 420
SP|P23475|XRCC6_MOUSE YLRPSLFVYPEESLVSGSSTLFSALLTKCVEKKVIAVCRYTPRKNVSPYFVALVPQEEEL 418
                      *************** ********** **:**:* *:******:*: *************

SP|P12956|XRCC6_HUMAN DDQKIQVTPPGFQLVFLPFADDKRKMPFTEKIMATPEQVGKMKAIVEKLRFTYRSDSFEN 480
SP|P23475|XRCC6_MOUSE DDQNIQVTPGGFQLVFLPYADDKRKVPFTEKVTANQEQIDKMKAIVQKLRFTYRSDSFEN 478
                      ***:***** ********:******:*****: *. **:.******:*************

SP|P12956|XRCC6_HUMAN PVLQQHFRNLEALALDLMEPEQAVDLTLPKVEAMNKRLGSLVDEFKELVYPPDYNPEGKV 540
SP|P23475|XRCC6_MOUSE PVLQQHFRNLEALALDMMESEQVVDLTLPKVEAIKKRLGSLADEFKELVYPPGYNPEGKV 538
                      ****************:** **.**********::******.**********.*******

SP|P12956|XRCC6_HUMAN TKRKHDNEGSGSKRPKVEYSEEELKTHISKGTLGKFTVPMLKEACRAYGLKSGLKKQELL 600
SP|P23475|XRCC6_MOUSE AKRKQDDEGSTSKKPKVELSEEELKAHFRKGTLGKLTVPTLKDICKAHGLKSGPKKQELL 598
                      :***:*:*** **:**** ******:*: ******:*** **: *:*:***** ******

SP|P12956|XRCC6_HUMAN EALTKHFQD- 609
SP|P23475|XRCC6_MOUSE DALIRHLEKN 608
                      :** :*::.
```

Supplementary Figure 1

Candidate phosphorylation sites of Ku70 that might influence Ku70-DNA interaction

Candidate phosphorylation sites expected from Figure 1a are indicated in homology alignment between human and mouse Ku70 amino acid sequences. Ser77 and Ser78 are colored red, and Thr90, Thr401 and Ser520 are indicated blue.

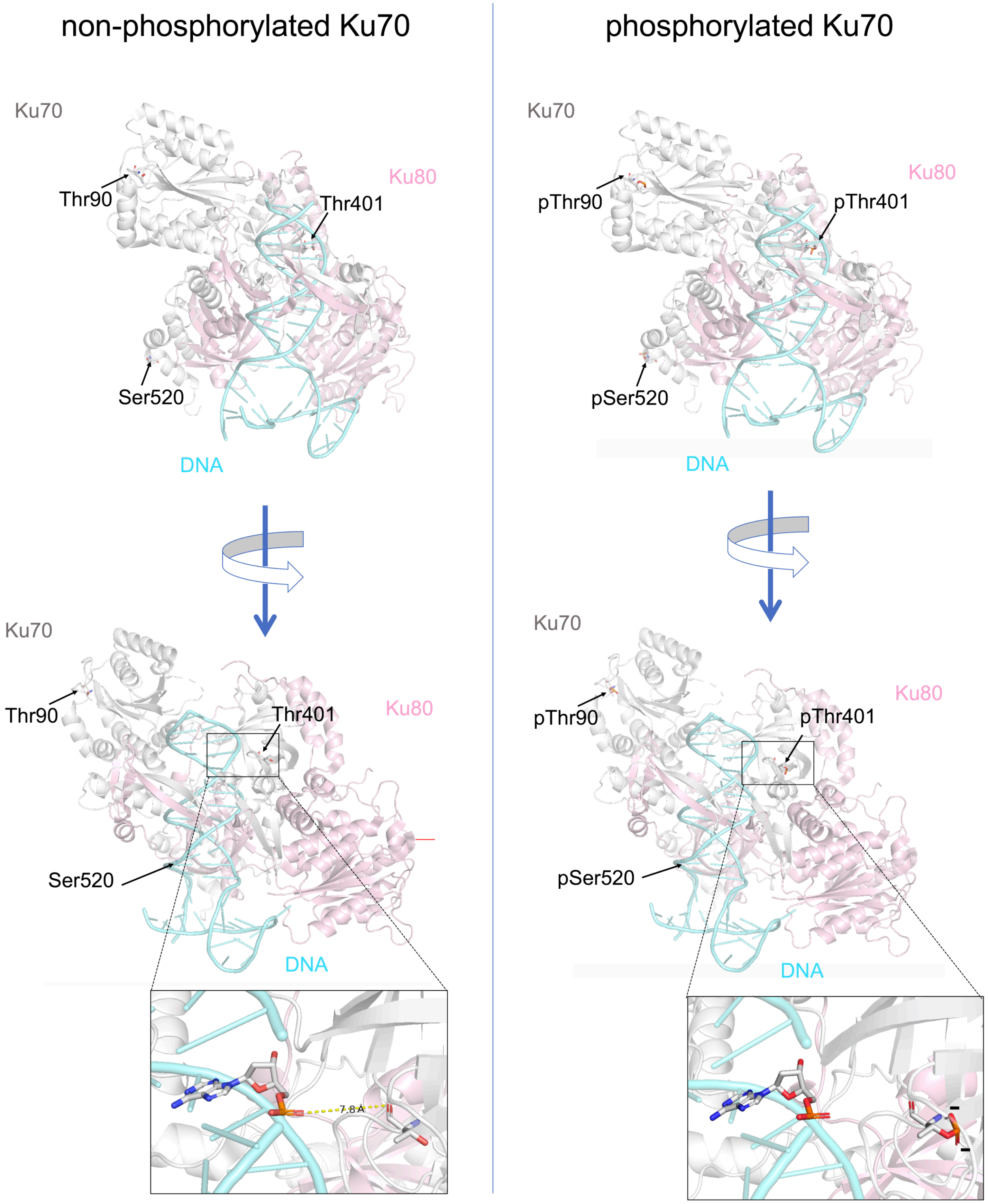

Supplementary Figure 2

Candidate phosphorylation sites in the model of Ku70 by PyMOL

Based on Ku70 structure at PDB (ID: 1JEY) (left), structure of Ku70 was modeled by PyMOL (Schrödinger, LLC) (right). The positions of Thr90, Thr401 and Ser520 are indicated in the complex of non-phosphorylated (left) or phosphorylated (right) Ku70 with Ku80 and double strand DNA. They are not sufficient for affecting the interaction surface of Ku70 and DNA or of Ku70 and Ku80. Inlays show the region around Thr401 of non-phosphorylated (left) or phosphorylated (right).

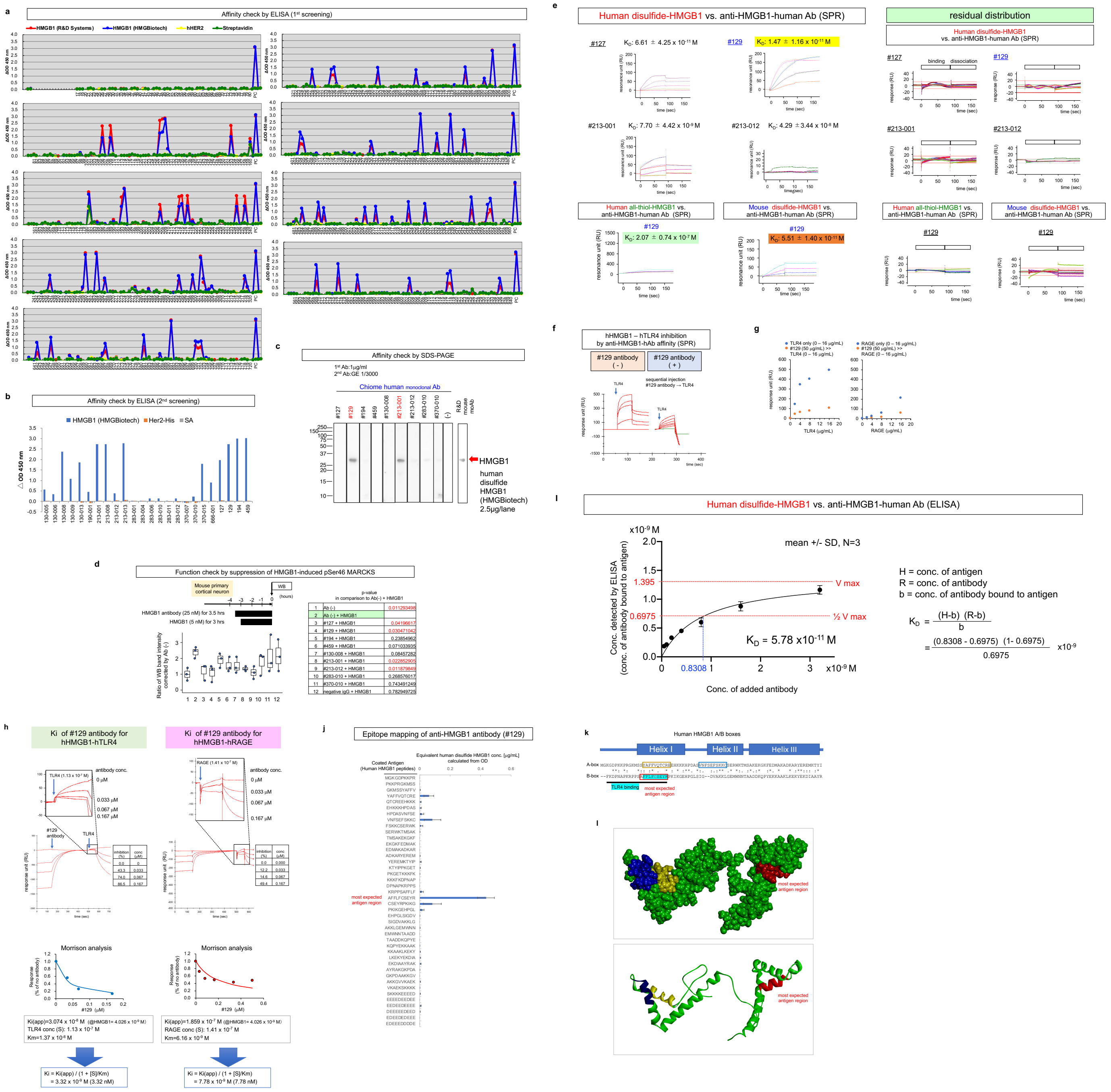

Supplementary Figure 3

Generation and characterization of human monoclonal anti-HMGB1 antibody

a) Human monoclonal HMGB1 antibodies were screened for their affinities to human disulfide HMGB1 via an ELISA. The clone numbers are indicated along the x-axis. The primary antibody was the Chiome human monoclonal anti-HMGB1 antibody. The secondary antibody was goat anti-mouse IgG-Fc fragment HRP-conjugated (Bethyl Laboratories, Montgomery, TX, USA; A90-131P). In the positive control (PC), human monoclonal anti-HMGB1 antibody (R&D Systems, Minneapolis, MN, USA; Cat# MAB1690; mouse IgG2b) was used as the positive control.
b) Second screening by ELISA.
c) The affinities of the human monoclonal anti-HMGB1 antibodies to human disulfide HMGB1 were assessed via SDS-PAGE. Antibodies #129 and #213-001 showed specific bands, while the other antibodies with high affinities to human disulfide HMGB1 as detected via the SPR analysis in Supplementary Figure 3e (#127 and #213-012) did not bind to human disulfide HMGB1 in the SDS-PAGE experiment, suggesting that these antibodies might recognize specific structures rather than specific sequences of human HMGB1.
d) Functional screening of the candidate human monoclonal anti-HMGB1 antibodies. The ratios of suppression of the HMGB1-induced increase in the pSer46-MARCKS level in primary cultured mouse cortical neurons after addition of human monoclonal anti-HMGB1 antibody at 3.75 μg/mL (25 nM) were calculated from the band signal intensities obtained via a western blot analysis. The pSer46-MARCKS band signal intensity was standardized to the intensity of the beta-actin signal with the same filter and then corrected by the mean value of lane 1.
e) Candidate antibodies, generated by the ADLib system followed by nucleotide sequence optimization for efficient expression in HEK and CHO cells, were examined for their binding affinities to human disulfide-HMGB1. In the case of the best candidate antibody (#129), the affinities to human all-thiol-HMGB1 and mouse disulfide-HMGB1 were examined by SPR analysis.
f) The inhibitory effect of human monoclonal anti-HMGB1 antibody #129 on the interaction between chip-immobilized human disulfide HMGB1 and TLR4 in flow at multiple concentrations.
g) Addition of antibody #129 suppressed the HMGB1-TLR4 interaction to less than 20% at all TLR4 concentrations. Similarly, antibody

#129 suppressed the HMGB1-RAGE interaction to about 30%.
h) The inhibitory effect of human monoclonal anti-HMGB1 antibody (#129) on the interaction between immobilized human disulfide HMGB1 and human TLR4 in flow at a fixed concentration. The SPR analysis showed that the antibody inhibited the HMGB1-TLR4 and HMGB1-RAGE interactions in a concentration-dependent manner. The lower calculations show the Ki values of antibody #129 for the HMGB1-TLR4 and HMGB1-RAGE interactions.
i) ELISA-based analysis of K_D value of human monoclonal anti-HMGB1 antibody #129 to human dsHMGB1. Method to calculate K_D value from Michaelis–Menten equation is shown.
j) ELISA-based epitope mapping with synthetic peptides covering the entire human HMGB1 amino acid sequence. Neighboring peptides were designed to overlap by 50%. The OD of each well was measured, and the equivalent HMGB1 concentration was calculated from a standard curve. The bar graph shows the mean ± s.e.m. (n = 4).
k) HMGB1 possesses two HMG boxes, each of which contains three α-helixes that are used for binding to the minor grove of the DNA double helix. An alignment of the amino acid sequences of the three α-helixes of HMG box A and B is shown. The TLR4-binding site overlaps with the major epitope located in the first α-helix (helix I) of HMG box B, and the minor epitope is located in the first α-helix of HMG box A.
l) The major and minor epitopes are indicated with red and yellow/blue colors on a three-dimensional structure of human HMGB1. The coordinates for the NMR solution structures were from Protein Data Bank entry ID 2YRQ. The figures were created with BIOVIA Discovery Studio (Dassault Systèmes).
Box plots show the median, quartiles and whiskers that represent data outside 25th to 75th percentile range.

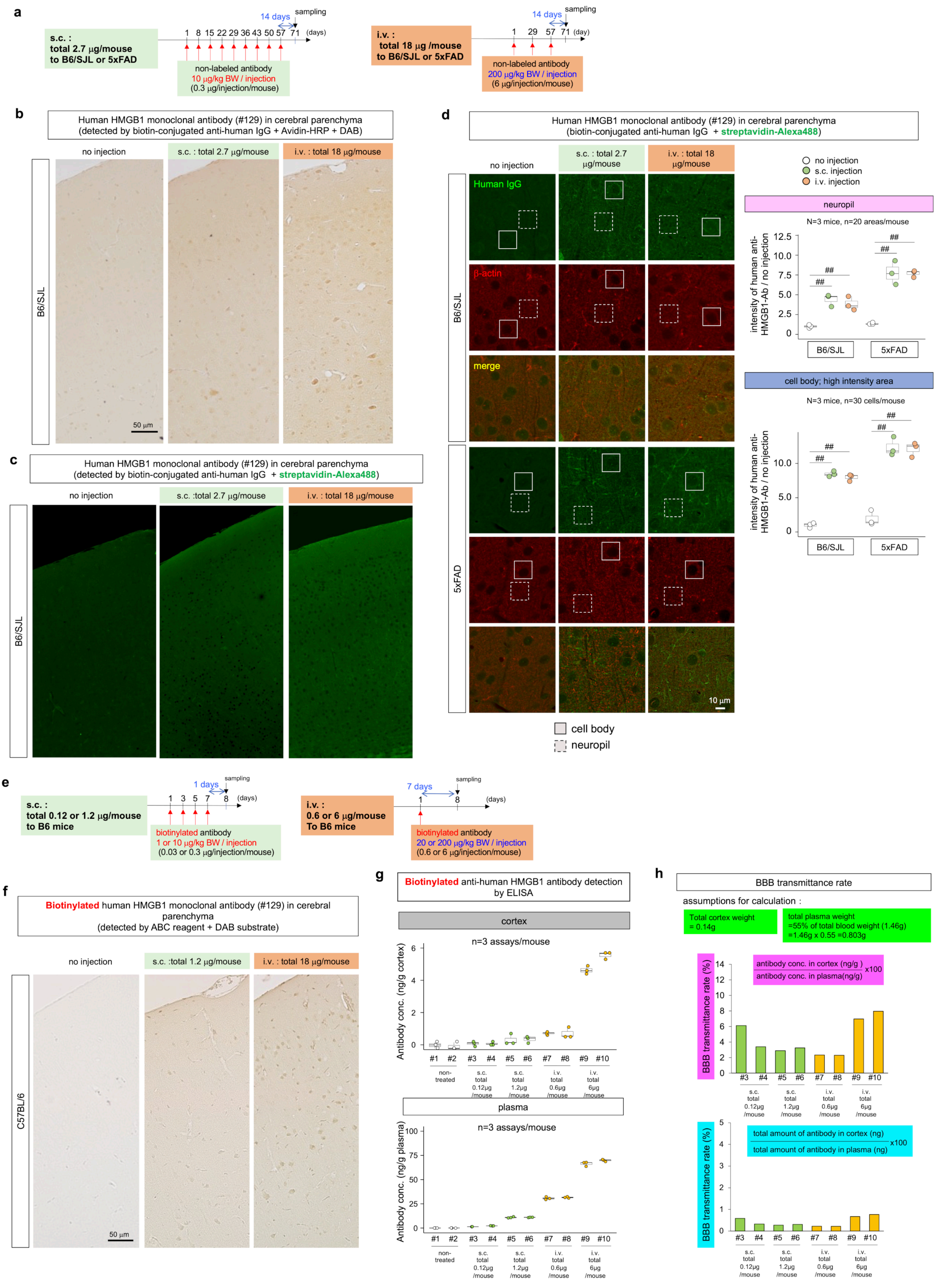
a
s.c. : total 2.7 μg/mouse to B6/SJL or 5xFAD
14 days
sampling
1 8 15 22 29 36 43 50 57 71 (days)
non-labeled antibody
10 μg/kg BW / injection
(0.3 μg/injection/mouse)
i.v. : total 18 μg /mouse to B6/SJL or 5xFAD
1 29 57 71 (day)
non-labeled antibody
200 μg/kg BW / injection
(6 μg/injection/mouse)
b
Human HMGB1 monoclonal antibody (#129) in cerebral parenchyma
(detected by biotin-conjugated anti-human IgG + Avidin-HRP + DAB)
no injection
s.c. : total 2.7 μg/mouse
i.v. : total 18 μg/mouse
B6/SJL
50 μm
c
Human HMGB1 monoclonal antibody (#129) in cerebral parenchyma
(detected by biotin-conjugated anti-human IgG + streptavidin-Alexa488)
d
Human HMGB1 monoclonal antibody (#129) in cerebral parenchyma
(biotin-conjugated anti-human IgG + streptavidin-Alexa488)
no injection
s.c. : total 2.7 μg/mouse
i.v. : total 18 μg/mouse
Human IgG
β-actin
merge
5xFAD
10 μm
cell body
neuropil
no injection
s.c. injection
i.v. injection
neuropil
N=3 mice, n=20 areas/mouse
intensity of human anti-HMGB1-Ab / no injection
##
B6/SJL
5xFAD
cell body; high intensity area
N=3 mice, n=30 cells/mouse
e
s.c. : total 0.12 or 1.2 μg/mouse to B6 mice
1 days
1 3 5 7 8 (days)
biotinylated antibody
1 or 10 μg/kg BW / injection
(0.03 or 0.3 μg/injection/mouse)
i.v. : 0.6 or 6 μg/mouse To B6 mice
7 days
biotinylated antibody
20 or 200 μg/kg BW / injection
(0.6 or 6 μg/injection/mouse)
f
Biotinylated human HMGB1 monoclonal antibody (#129) in cerebral parenchyma
(detected by ABC reagent + DAB substrate)
no injection
s.c. :total 1.2 μg/mouse
i.v. : total 18 μg/mouse
C57BL/6
50 μm
g
Biotinylated anti-human HMGB1 antibody detection by ELISA
cortex
n=3 assays/mouse
Antibody conc. (ng/g cortex)
#1 #2 #3 #4 #5 #6 #7 #8 #9 #10
non-treated
s.c. total 0.12μg /mouse
s.c. total 1.2μg /mouse
i.v. total 0.6μg /mouse
i.v. total 6μg /mouse
plasma
Antibody conc. (ng/g plasma)
h
BBB transmittance rate
assumptions for calculation :
Total cortex weight = 0.14g
total plasma weight =55% of total blood weight (1.46g) =1.46g x 0.55 =0.803g
BBB transmittance rate (%)
antibody conc. in cortex (ng/g) / antibody conc. in plasma(ng/g) x100
total amount of antibody in cortex (ng) / total amount of antibody in plasma (ng) x100

Supplementary Figure 4
Human monoclonal anti-HMGB1 antibody passes through the blood-brain barrier
a) Protocols for the subcutaneous and intravenous injection of human monoclonal anti-HMGB1 antibody (#129) to 5xFAD mice. The samples were used for analyses in b - d.
b) Immunohistochemistry detected human monoclonal anti-HMGB1 antibody (#129) in brain tissues by biotin-conjugated anti-human IgG antibody followed by detection with Avidin-HRP and DAB.
c) Immunohistochemistry detected human monoclonal anti-HMGB1 antibody (#129) in brain tissues by biotin-conjugated anti-human IgG antibody followed by streptavidin-Alexa488.
d) Left panels show high magnification of immunostains of human monoclonal anti-HMGB1 antibody (#129) shown in C. Right graphs show quantification of signal intensities of cell body and neuropils.
e) Protocols for the subcutaneous and intravenous injection of biotinylated human monoclonal anti-HMGB1 antibody (#129) into C57BL/6 mice, whose samples were used for analyses in b - d.
f) Biotinylated human monoclonal anti-HMGB1 antibody (#129) in the brain tissues was detected by ABC reagent and DAB.
g) The amounts of human monoclonal anti-HMGB1 antibody (#129) were determined by ELISA in cerebral cortex tissue and plasma samples from C57BL/6 mice (#1 – #10) after subcutaneous (s.c.) or intravenous (i.v.) injections of different doses of #129 as described above in (e).
h) BBB transmittance rate was estimated under two conditions.
Box plots show the median, quartiles and whiskers that represent data outside 25th to 75th percentile range.

a

Summary of identified peptides

Month		1			3			6		
Experiment		#1	#2	#3	#1	#2	#3	#1	#2	#3
Confidence	Total / Phospho-									
>95%	Total	62167	101192	110725	84986	63419	52140	63575	57995	48003
	Phospho-	13041	18306	26616	30016	18443	18257	19464	24741	16810
>90%	Total	66562	106723	117649	88664	77718	56143	67760	60567	51357
	Phospho-	13689	19151	28001	30973	21299	19337	20545	25450	17770
>66%	Total	78187	118580	132761	96854	85892	65989	78446	66293	59579
	Phospho-	16176	21073	32027	33542	23608	22660	24043	27821	20761

Summary of identified proteins

Month		1			3			6		
Experiment		#1	#2	#3	#1	#2	#3	#1	#2	#3
Confidence										
>95%	Total proteins	1944	2125	2642	2039	1734	1640	1738	1500	1801
>90%		2054	2235	2780	2129	2020	1669	1764	1572	1823
>66%		2456	2502	3068	2317	2268	1671	1764	1727	1823

b

Pathological Network of Phosphoproteins in 5xFAD based on PPI databases
(changed phosphopeptides at q<0.05 in Welch's test with post-hoc BH procedure)

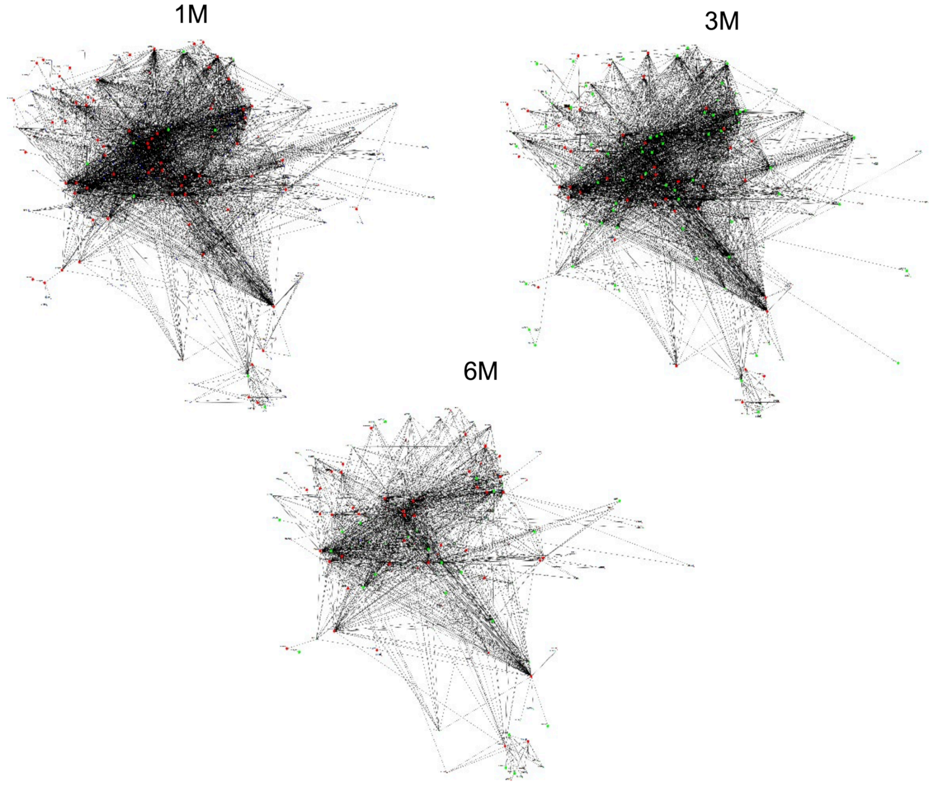

URL: http://suppl.atgc.info/031/

Supplementary Figure 5
Pathological molecular network of phosphoproteins in 5xFAD mouse

a) Summary of the identified peptides and proteins at various confidence levels. In the case of peptides, the numbers reflect redundant counting of overlapping peptides.

b) Molecular network of pathologically altered phosphoproteins in 5xFAD mice at 1, 3, and 6 months of age in comparison with non-Tg sibling mice (B6/SJL). To establish whether a protein was changed, q-values were calculated from p-values in comparisons between groups using the Benjamini-Hochberg procedure. The changed proteins were used as nodes and mapped onto the integrated protein-protein interaction (PPI) database supplied by the Genome Network Platform of the National Institute of Genetics (http://genomenetwork.nig.ac.jp/index_e.html), which includes the experimentally supported PPI databases of the Human Genome Project (GNP) and databases from BIND (http://www.bind.ca/), BioGrid (http://www.thebiogrid.org/), HPRD (http://www.hprd.org/), IntAct (http://www.ebi.ac.uk/intact/site/index.jsf), and MINT (http://mint.bio.uniroma2.it/mint/Welcome.do). The edges were allowed to connect one additional node even if that node was not changed or detected in our phosphoproteome analysis.

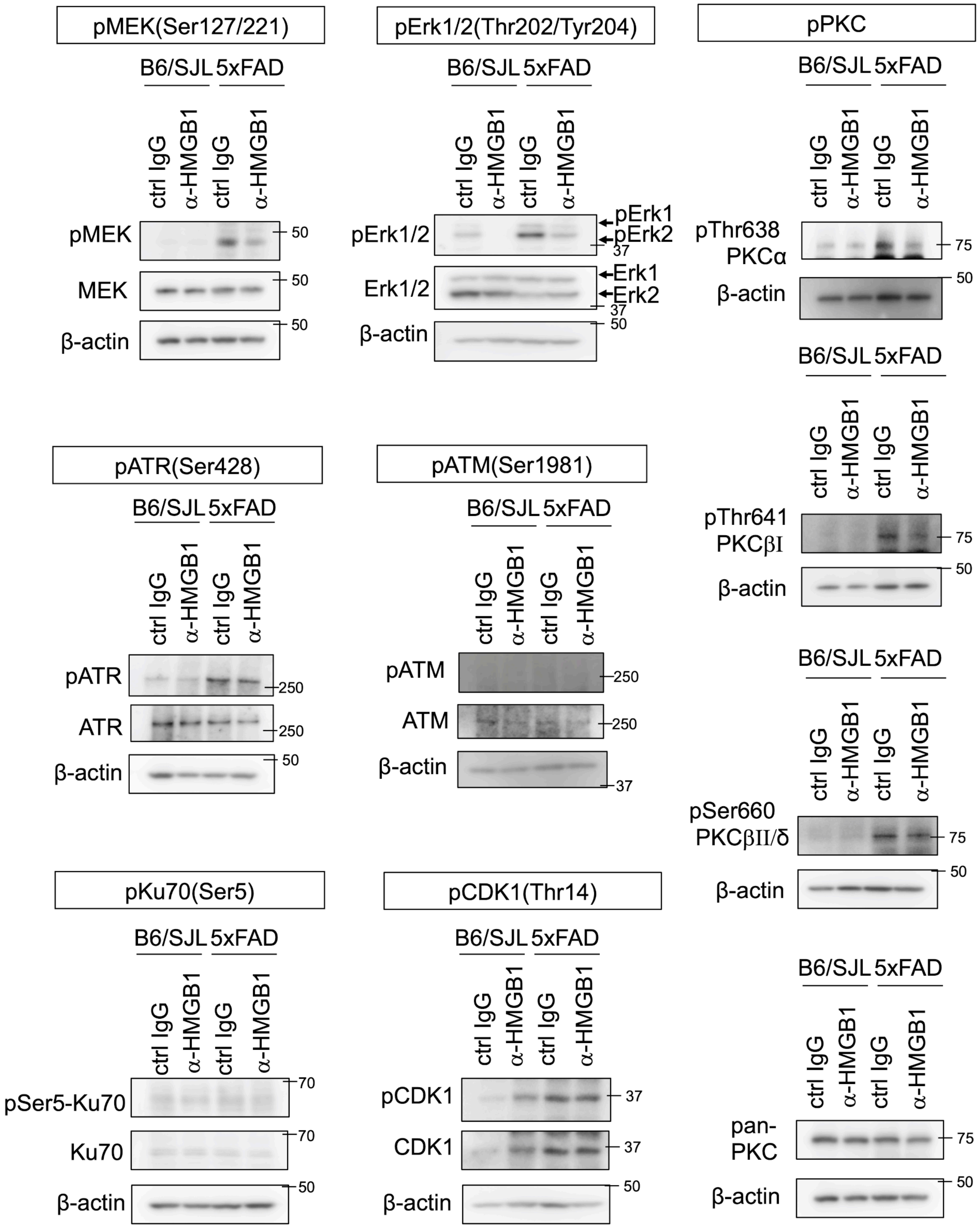

Supplementary Figure 6

Activated kinases in the downstream of HMGB1-triggered signaling pathway

Western blot analysis of candidate kinases of Ku70 (MEK, ERK, PKCs, CDK1, ATR, ATM) to examine their activation in the 5xFAD AD model mice and the rescue by human monoclonal anti-HMGB1 antibody (#129). MEK, ERK, and PKCs (especially PKCα) were activated in 5xFAD mice and rescued human monoclonal anti-HMGB1 antibody.

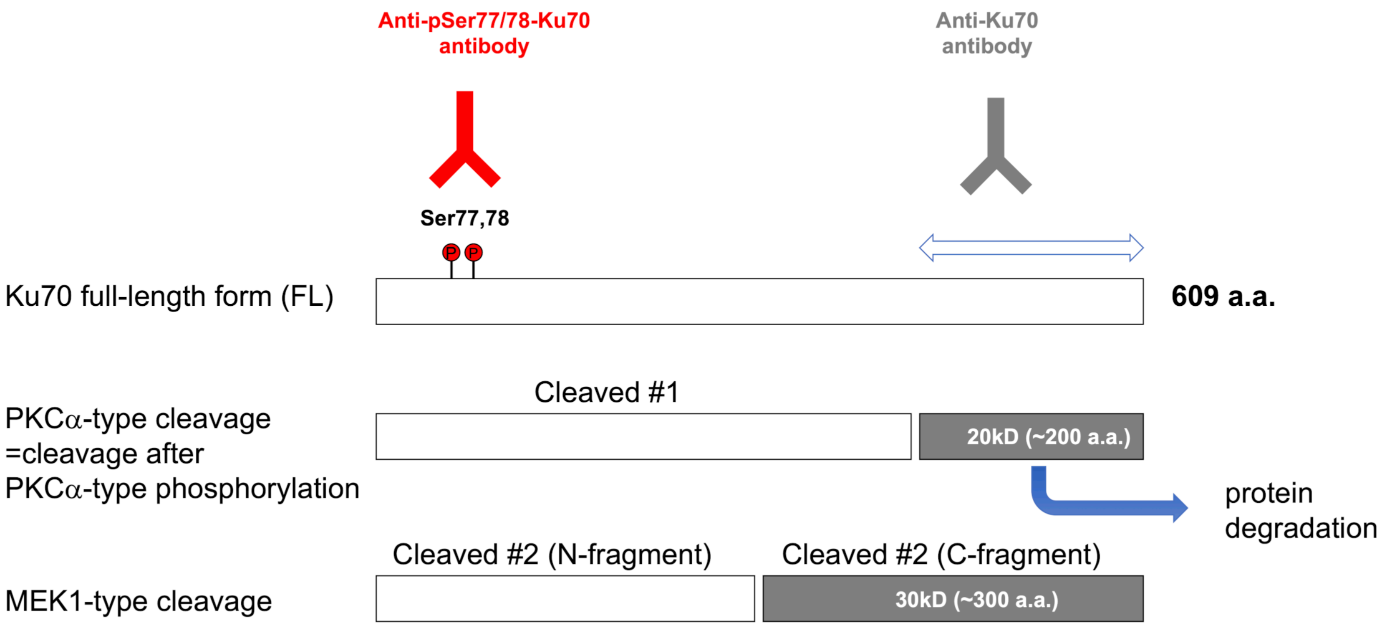

Supplementary Figure 7
Protein cleavage of Ku70 after phosphorylation
In vitro phosphorylation experiments revealed that Ku70 was cleaved after phosphorylation at Ser77/78 by PKCα and MEK1 and that the cleavage sites were different in PKCα-type and MEK1-type phosphorylation (Figure 5a) presumably because other additional phosphorylation sites of Ku70 were different in two kinases (Supplementary Figure 5a). The scheme here explains well that the band patterns in western blots (Figure 5d) are consistent with the cleavage after phosphorylation at Ser77/78.

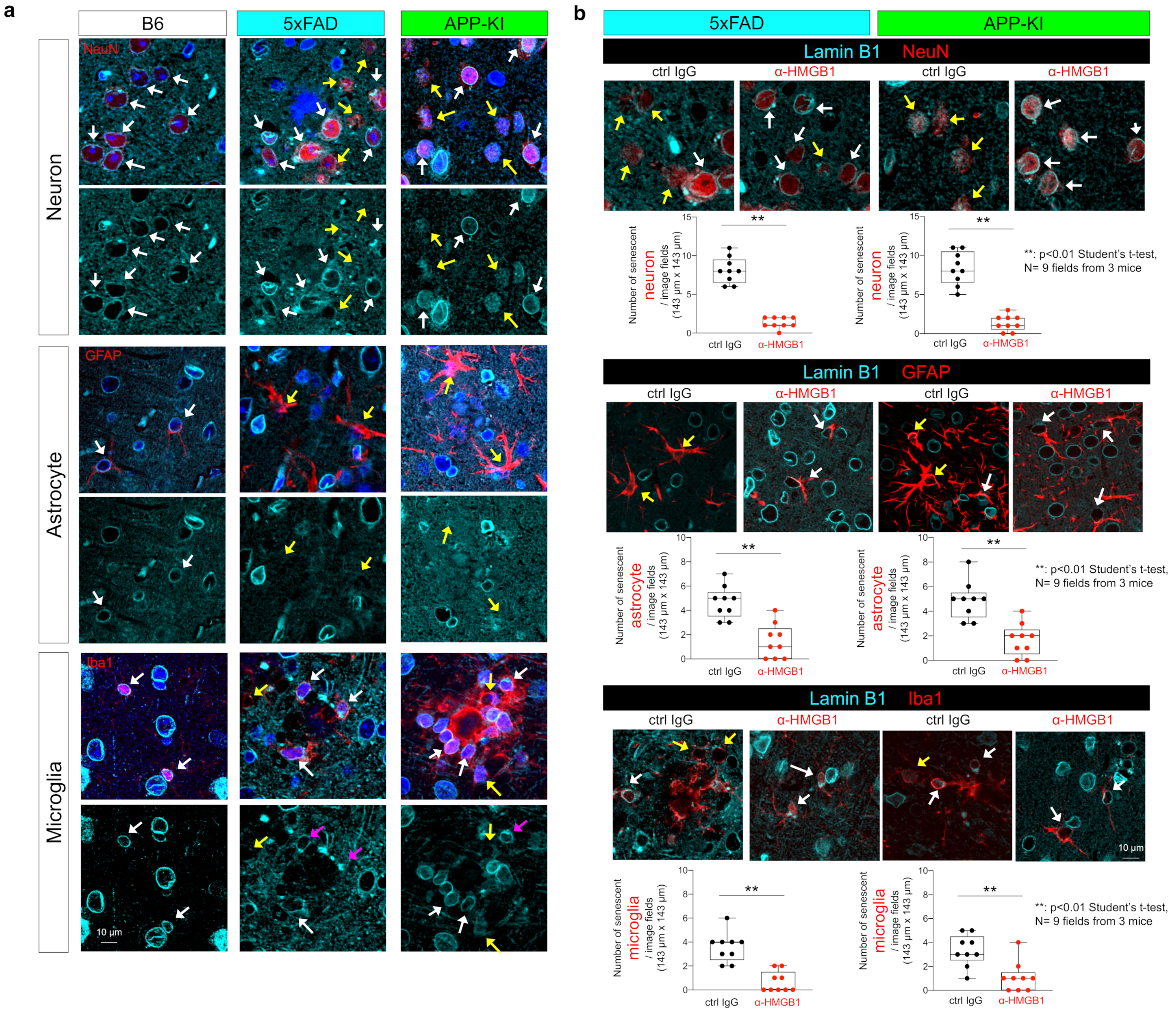

Supplementary Figure 8

Senescence of neurons and glia in AD model mice and the recovery by anti-HMGB1 antibody treatment

a) Normal neuron, astrocyte and microglia of C57BL/6 mice (B6), which are stained with NeuN, GFAP and Iba1 respectively (white arrow), show the ring-like stains of a nuclear membrane protein Lamin B1 as a senescence marker. The ring of Lamin B1 becomes unclear or disappears in a part of neurons and astrocytes of 5xFAD or APP-KI mice (yellow arrow). In microglia of 5xFAD or APP-KI mice, only a few cells lost the Lamin ring while some cells contains an abnormal focus (magenta arrow).

b) Intravenous administration of human monoclonal anti-HMGB1 antibody (#129) reduced the number of senescent neurons, astrocytes and microglia in the cerebral cortex of 5xFAD and APP-KI mice.

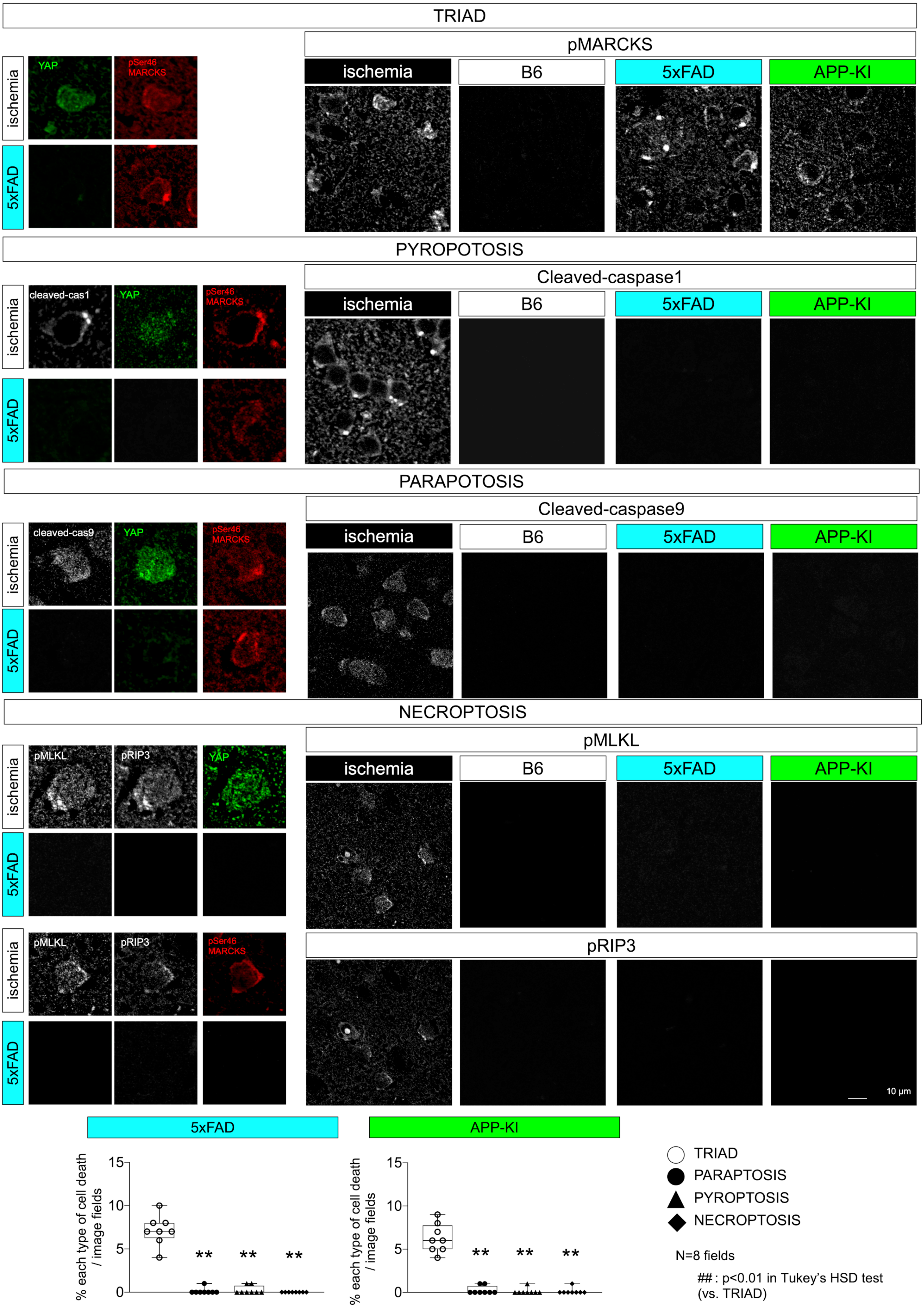

TRIAD
pMARCKS
ischemia
5xFAD
YAP
pSer46 MARCKS
ischemia
B6
5xFAD
APP-KI
PYROPOTOSIS
Cleaved-caspase1
cleaved-cas1
YAP
pSer46 MARCKS
PARAPOTOSIS
Cleaved-caspase9
cleaved-cas9
NECROPTOSIS
pMLKL
pRIP3
10 μm
5xFAD
APP-KI
% each type of cell death / image fields
15
10
5
0
**
TRIAD
PARAPTOSIS
PYROPTOSIS
NECROPTOSIS
N=8 fields
##: p<0.01 in Tukey's HSD test (vs. TRIAD)

Supplementary Figure 9
Identification of necrosis as TRIAD

To identify the type of the late-onset necrosis in AD mouse models, dying cells were detected by their specific markers for TRIAD or other types of necrosis in the cerebral cortex of 5xFAD, APP-KI or background mice at 8 months of age. Cerebral cortex after ischemia by occlusion of bilateral common carotid arteries was used as positive controls for all types of necrosis. Lower graphs show the number of dying cells in the visual field (143 μm x143 μm) under each type of necrosis.

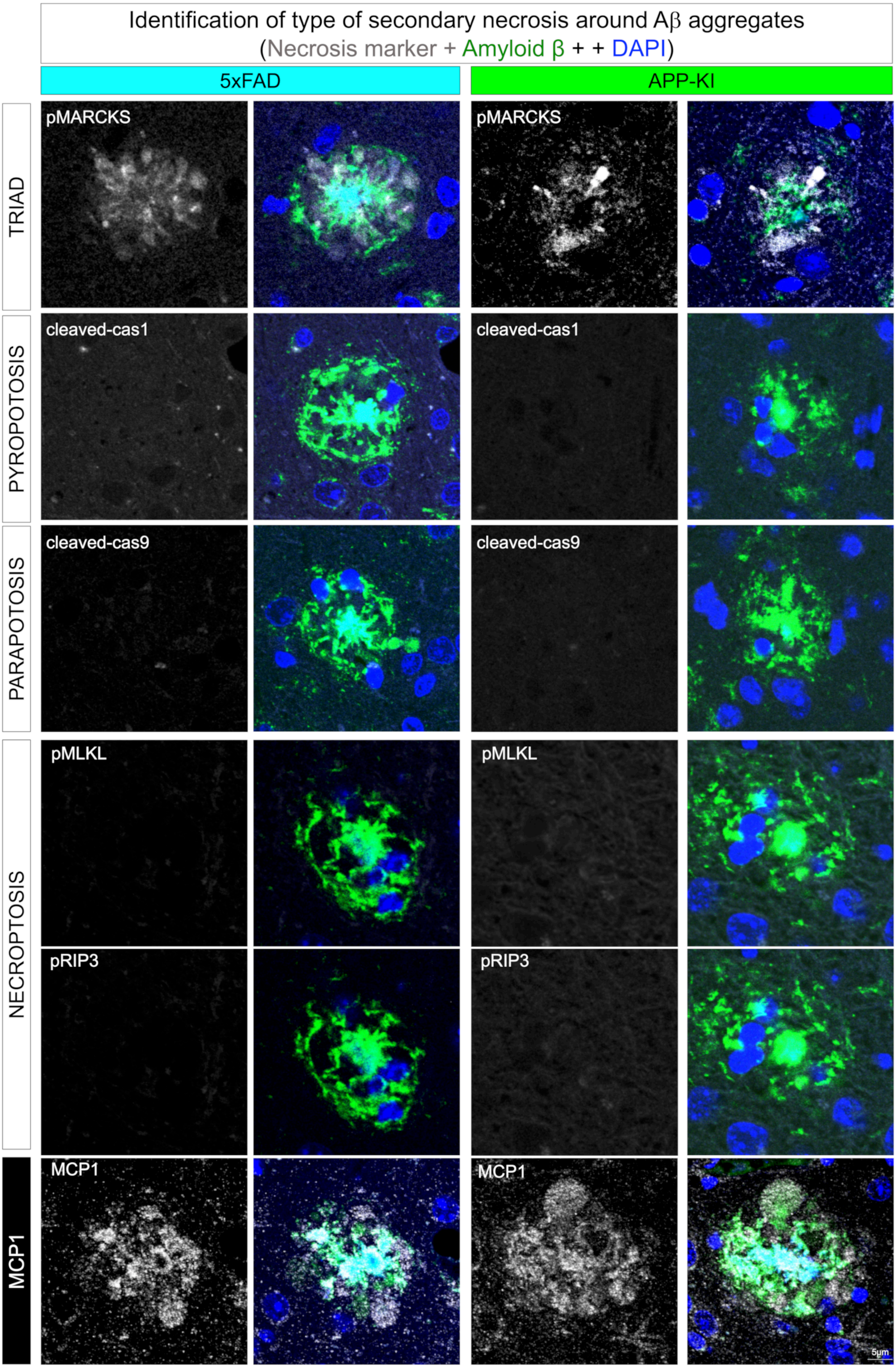
Identification of type of secondary necrosis around Aβ aggregates
(Necrosis marker + Amyloid β + + DAPI)
5xFAD
APP-KI
TRIAD
PYROPTOSIS
PARAPTOSIS
NECROPTOSIS
MCP1
pMARCKS
cleaved-cas1
cleaved-cas9
pMLKL
pRIP3
MCP1
5μm

Supplementary Figure 10

Identification of secondary necrosis around extracellular Aβ aggregates as TRIAD

We analyzed secondary cell death around extracellular Aβ aggregates by necrosis markers. TRIAD was detected almost exclusively among various types of necrosis. MCP1 was used as a positive control.

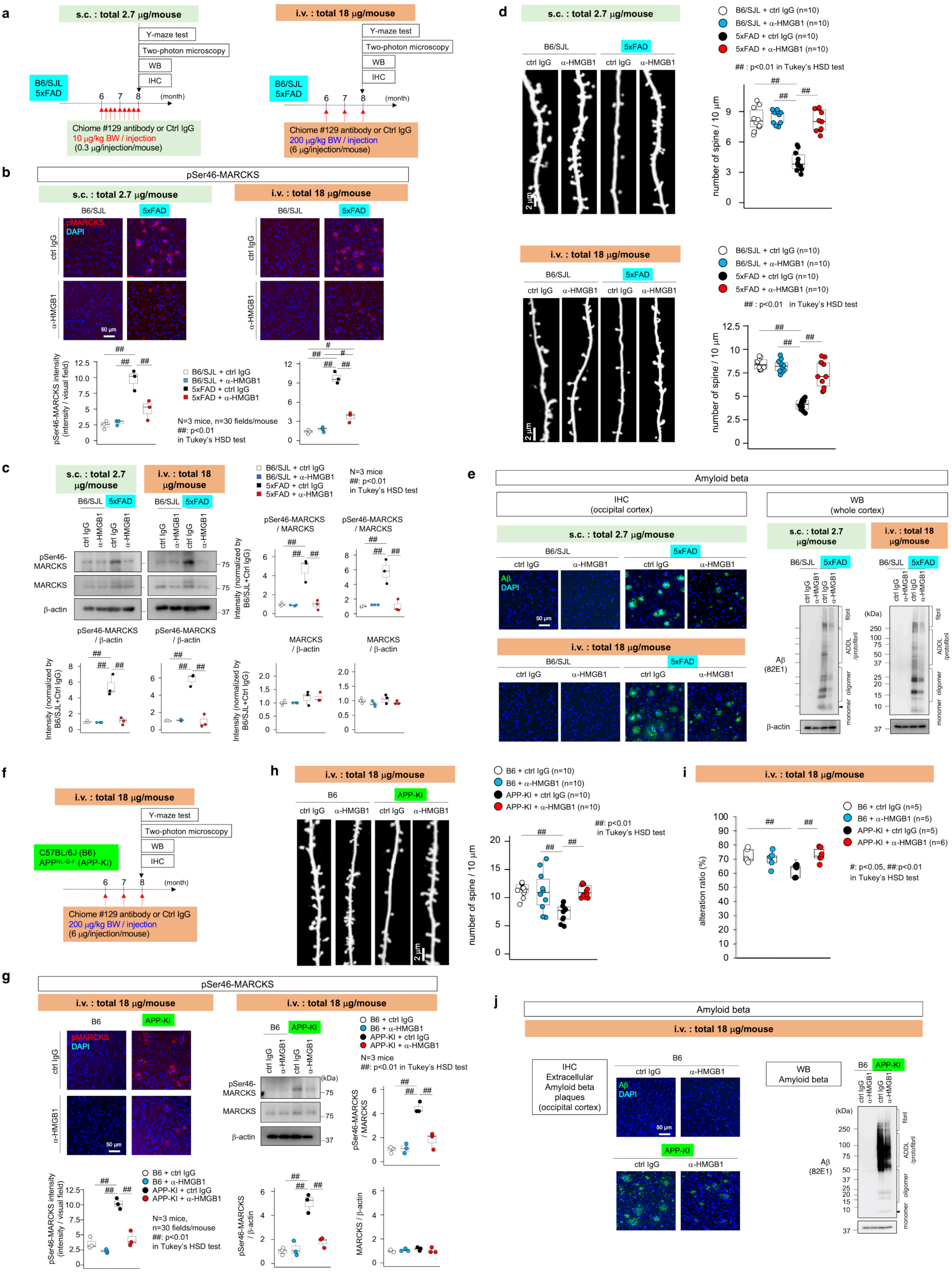

a
s.c. : total 2.7 μg/mouse
i.v. : total 18 μg/mouse
Y-maze test
Two-photon microscopy
WB
IHC
B6/SJL
5xFAD
(month)
Chiome #129 antibody or Ctrl IgG
10 μg/kg BW / injection
(0.3 μg/injection/mouse)
200 μg/kg BW / injection
(6 μg/injection/mouse)
b
pSer46-MARCKS
pMARCKS
DAPI
ctrl IgG
α-HMGB1
50 μm
pSer46-MARCKS intensity (intensity / visual field)
B6/SJL + ctrl IgG
B6/SJL + α-HMGB1
5xFAD + ctrl IgG
5xFAD + α-HMGB1
N=3 mice, n=30 fields/mouse
##: p<0.01
in Tukey's HSD test
c
pSer46-MARCKS
MARCKS
β-actin
pSer46-MARCKS / MARCKS
pSer46-MARCKS / β-actin
MARCKS / β-actin
Intensity (normalized by B6/SJL+Ctrl IgG)
N=3 mice
##: p<0.01
in Tukey's HSD test
d
B6/SJL + ctrl IgG (n=10)
B6/SJL + α-HMGB1 (n=10)
5xFAD + ctrl IgG (n=10)
5xFAD + α-HMGB1 (n=10)
: p<0.01 in Tukey's HSD test
number of spine / 10 μm
2 μm
e
Amyloid beta
IHC
(occipital cortex)
WB
(whole cortex)
Aβ
DAPI
Aβ
(82E1)
fibril
ADDL /protofibril
oligomer
monomer
(kDa)
f
C57BL/6J (B6)
APP^NL-G-F (APP-KI)
g
B6 + ctrl IgG
B6 + α-HMGB1
APP-KI + ctrl IgG
APP-KI + α-HMGB1
N=3 mice, n=30 fields/mouse
##: p<0.01 in Tukey's HSD test
h
B6 + ctrl IgG (n=10)
B6 + α-HMGB1 (n=10)
APP-KI + ctrl IgG (n=10)
APP-KI + α-HMGB1 (n=10)
i
B6 + ctrl IgG (n=5)
B6 + α-HMGB1 (n=5)
APP-KI + ctrl IgG (n=5)
APP-KI + α-HMGB1 (n=6)
#: p<0.05, ##:p<0.01
in Tukey's HSD test
alteration ratio (%)
j
IHC
Extracellular
Amyloid beta
plaques
(occipital cortex)
WB
Amyloid beta

Supplementary Figure 11
Interruption of HMGB1-signal ameliorates post-onset AD model mice

a) Protocols for the subcutaneous and intravenous injection of human monoclonal anti-HMGB1 antibody (#129) into 5xFAD mice. The subcutaneous injection experiment is similar to that in Figure 3a (10 μg/kg of BW).

b) Immunohistochemical staining of cerebral cortex (occipital cortex) samples with anti-pSer46-MARCKS antibody. 5xFAD mice showed an increase in the number of degenerative neurites surrounding extracellular Aβ plaques, and their number was substantially reduced by treatment with human monoclonal anti-HMGB1 antibody. Control IgG-treated 5xFAD mice and HMGB1 antibody-treated/control IgG-treated non-transgenic sibling mice (B6/SJL) were similarly analyzed as controls. Representative images are shown in the left panels. The signal intensities per area (625 × 625 μm) were acquired from 10 visual fields for each mouse, and the average value was calculated. The values from three mice were used for the statistical analysis shown in the graphs on the right.

c) Western blot analysis with anti-pSer46-MARCKS antibody confirmed the increase of pSer46-MARCKS, but not total MARCKS, in whole cerebral cortex samples from 5xFAD mice. The increase was rescued via human monoclonal anti-HMGB1 antibody.

d) The effect of human monoclonal anti-HMGB1 antibody (#129) on dendritic spine density. In both types of administration, antibody #129 rescued the decrease of dendritic spine number observed in 5xFAD mice. No remarkable side effects on spine density of normal mice were observed.

e) The effect of human monoclonal anti-HMGB1 antibody (#129) on extracellular Aβ aggregates were investigated by immunohistochemistry (left panels) and by western blot analysis (right panels). In both types of administration, antibody #129 reduced Aβ burdens in brains of 5xFAD mice.

f) Protocols for intravenous injection of human monoclonal anti-HMGB1 antibody (#129) to APP-KI mice.

g) Left panels show immunohistochemistry of occipital cortex samples from APP-KI mice with anti-pSer46-MARCKS antibody. Right panels show western blot analysis of whole cortex from APP-KI mice with anti-pSer46-MARCKS antibody.

h) The effect of human monoclonal anti-HMGB1 antibody (#129) on dendritic spine density. In both types of administration, antibody #129 rescued the decrease of dendritic spine number observed in 5xFAD mice. No remarkable side effects on spine density of normal mice were observed.

i) Intravenous injection of human monoclonal anti-HMGB1 antibody (#129) to APP-KI mice recovered the alteration rate in a Y-maze test like in the case of 5xFAD mice shown in Figure 6e.

j) Staining of cerebral cortex samples with anti-Aβ antibody revealed the decrease of extracellular Aβ aggregates in APP-KI mice after administration of human monoclonal anti-HMGB1 antibody (#129). Right panels show western blots of whole cortex samples.

Box plots show the median, quartiles and whiskers that represent data outside 25th to 75th percentile range.

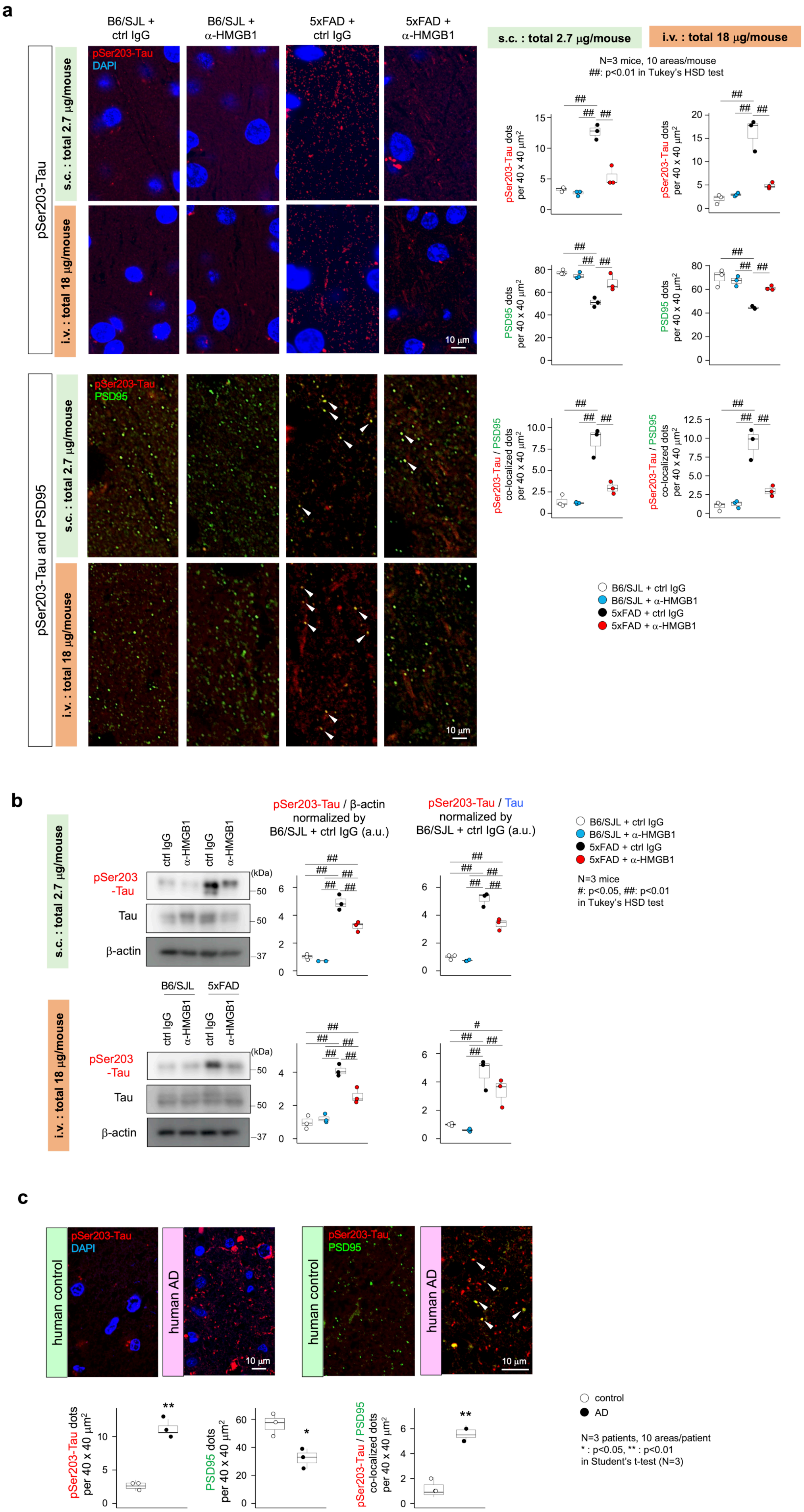
a
B6/SJL + ctrl IgG
B6/SJL + α-HMGB1
5xFAD + ctrl IgG
5xFAD + α-HMGB1
s.c. : total 2.7 μg/mouse
i.v. : total 18 μg/mouse
pSer203-Tau
pSer203-Tau
DAPI
10 μm
pSer203-Tau and PSD95
pSer203-Tau
PSD95
N=3 mice, 10 areas/mouse
##: p<0.01 in Tukey's HSD test
pSer203-Tau dots per 40 x 40 μm²
PSD95 dots per 40 x 40 μm²
pSer203-Tau / PSD95 co-localized dots per 40 x 40 μm²
B6/SJL + ctrl IgG
B6/SJL + α-HMGB1
5xFAD + ctrl IgG
5xFAD + α-HMGB1
b
pSer203 -Tau
Tau
β-actin
ctrl IgG
α-HMGB1
(kDa)
B6/SJL
5xFAD
pSer203-Tau / β-actin normalized by B6/SJL + ctrl IgG (a.u.)
pSer203-Tau / Tau normalized by B6/SJL + ctrl IgG (a.u.)
N=3 mice
#: p<0.05, ##: p<0.01
in Tukey's HSD test
c
human control
human AD
pSer203-Tau
DAPI
PSD95
10 μm
control
AD
pSer203-Tau dots per 40 x 40 μm²
PSD95 dots per 40 x 40 μm²
pSer203-Tau / PSD95 co-localized dots per 40 x 40 μm²
N=3 patients, 10 areas/patient
* : p<0.05, ** : p<0.01
in Student's t-test (N=3)

Supplementary Figure 12
Interruption of HMGB1-signal recovers synapse

a) Immunohistochemical staining of pSer-203Tau and PSD95 with occipital cortex tissues from 5xFAD mice and non-transgenic sibling mice (B6/SJL) after treatment of human monoclonal anti-HMGB1 antibody (#129) or control IgG according to the protocol shown in Figure 3a. Right graphs show the numbers of pSer203-tau, PSD95, and pSer203-tau/PSD95-colocalized dots in the four mouse groups.
b) A western blot showing the increased pSer203-tau abundance in 5xFAD mice and its rescue via treatment with human anti-HMGB1 antibody. Right graphs show quantitative analyses of the pSer203-tau signal intensities corrected by β-actin or tau.
c) Immunohistochemical staining of human postmortem brain tissue samples (occipital lobe) from patients with no neurological diseases (control) and AD patients with anti-pSer-203-tau and anti-PSD95 antibodies.
Box plots show the median, quartiles and whiskers that represent data outside 25th to 75th percentile range.

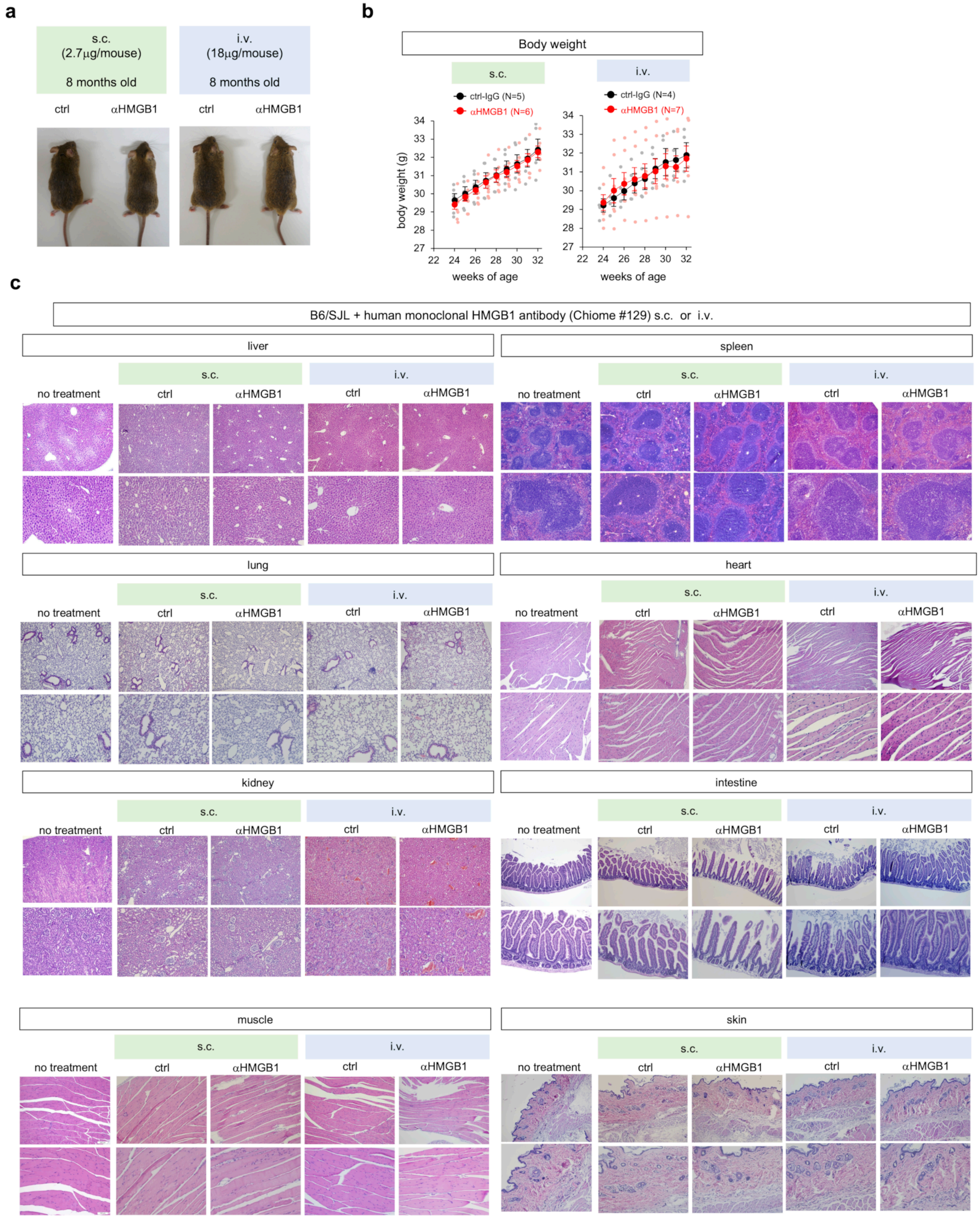

Supplementary Figure 13

Analysis of the side effects of subcutaneous and intravenous administration of human monoclonal anti-HMGB1 antibody

a) Appearance of mice receiving subcutaneous (2.7 μg/mouse) or intravenous (18 μg/mouse) administration of human monoclonal anti-HMGB1 antibody. Untreated mice were used as the first control, and mice receiving subcutaneous or intravenous administration of similar doses of human control IgG were used as the second control.

b) Body weight changes of mice that received human monoclonal anti-HMGB1 antibody (s.c. or i.v.) or control human IgG (as described above). Values in each group are summarized by mean ± S.E.M.

c) Hematoxylin-eosin staining of multiple organs prepared from mice that received s.c. or i.v. administration of human monoclonal anti-HMGB1 antibody or control human IgG.

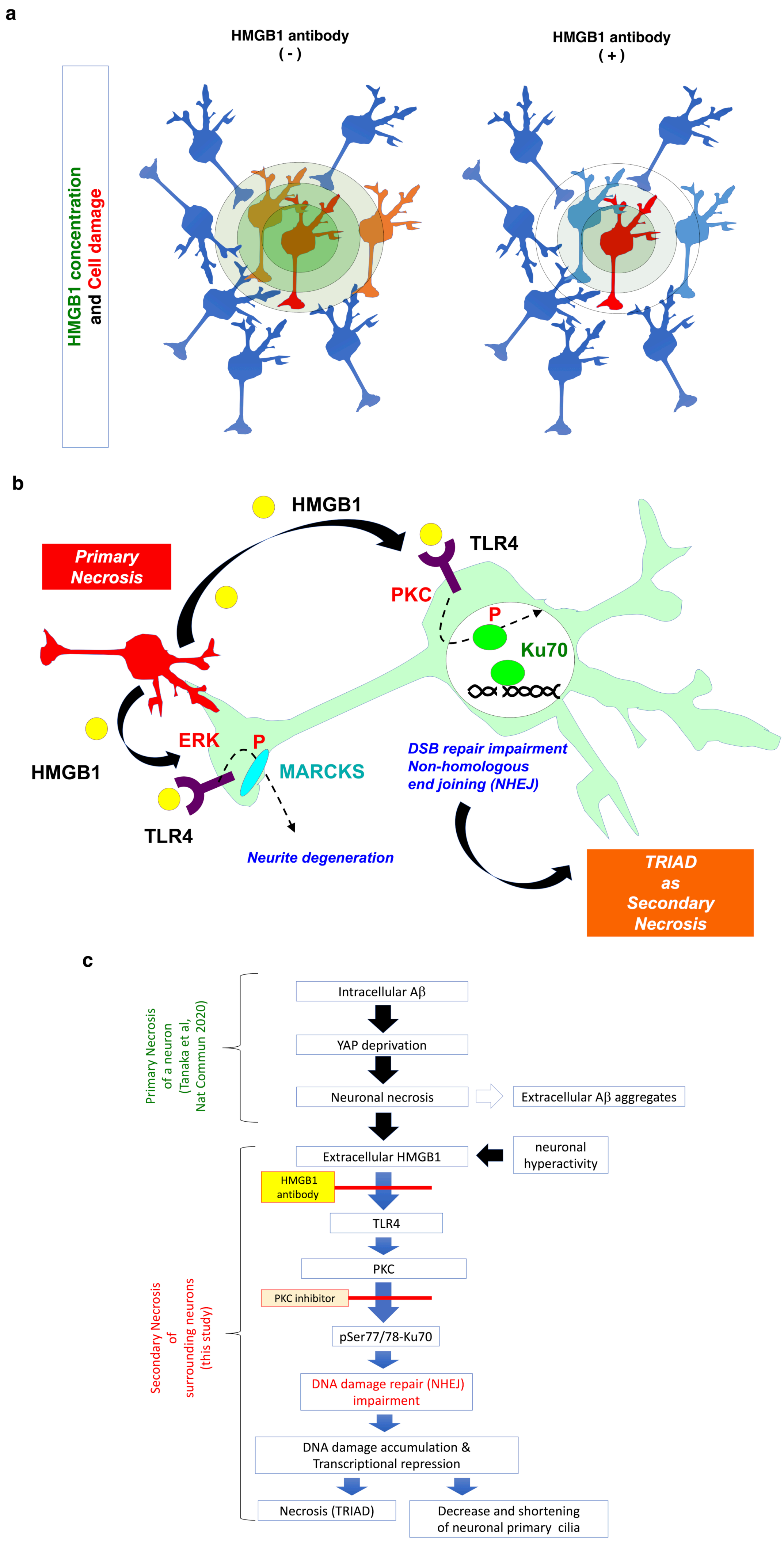
a
HMGB1 antibody (-)
HMGB1 antibody (+)
HMGB1 concentration and Cell damage
b
HMGB1
TLR4
PKC
P
Ku70
Primary Necrosis
ERK
P
MARCKS
HMGB1
TLR4
Neurite degeneration
DSB repair impairment
Non-homologous end joining (NHEJ)
TRIAD as Secondary Necrosis
c
Primary Necrosis of a neuron (Tanaka et al, Nat Commun 2020)
Intracellular Aβ
YAP deprivation
Neuronal necrosis
Extracellular Aβ aggregates
Extracellular HMGB1
neuronal hyperactivity
HMGB1 antibody
TLR4
PKC
PKC inhibitor
pSer77/78-Ku70
DNA damage repair (NHEJ) impairment
DNA damage accumulation & Transcriptional repression
Necrosis (TRIAD)
Decrease and shortening of neuronal primary cilia
Secondary Necrosis of surrounding neurons (this study)

Supplementary Figure 14
Expansion and suppression of neurodegeneration

a) Extracellular HMGB1 released from damaged neurons transfers the neurodegenerative stimulus to innocent surrounding neurons (left panel). Human monoclonal anti-HMGB1 antibody reduces the concentration of extracellular HMGB1 and suppresses HMGB1-induced expansion of neurodegenerative changes to neighboring neurons (right panel).

b) HMGB1 released from primary necrosis induces phosphorylation of Ku70 at Ser77/78 to impair DNA damage repair by non-homologous end joining (results in this paper) and phosphorylation of MARCKS to trigger neurite degeneration[38]. DNA damage accumulation leads to secondary necrosis.

c) Relationship between primary necrosis intrinsically caused by YAP deprivation by intracellular Aβ[37] and secondary necrosis extrinsically caused by HMGB1 and impairment of DNA damage repair after Ku70 phosphorylation (this study).
