# Supplementary material for: HMGB1 signaling phosphorylates Ku70 and impairs DNA damage repair in Alzheimer’s disease pathology
Source: Commun Biol. 2021 Oct 11;4:1175. doi: 10.1038/s42003-021-02671-4 (PMC8505418; doi:10.1038/s42003-021-02671-4)
Supplement: Supplementary file 3 — Description of Additional Supplementary Files [file 42003_2021_2671_MOESM3_ESM.pdf]

Description of Additional Supplementary Files

File name: Supplementary Data 1.
Description: All source data underling graphs and RNAseq in the main figures.
