# Supplementary material for: HMGB1 signaling phosphorylates Ku70 and impairs DNA damage repair in Alzheimer’s disease pathology
Source: Commun Biol. 2021 Oct 11;4:1175. doi: 10.1038/s42003-021-02671-4 (PMC8505418; doi:10.1038/s42003-021-02671-4)
Supplement: Supplementary file 5 — Reporting Summary [file 42003_2021_2671_MOESM5_ESM.pdf]

nature portfolio

Corresponding author(s): Hitoshi Okazawa

Last updated by author(s): Sep 4, 2021

Reporting Summary

Nature Portfolio wishes to improve the reproducibility of the work that we publish. This form provides structure for consistency and transparency in reporting. For further information on Nature Portfolio policies, see our Editorial Policies and the Editorial Policy Checklist.

Statistics

For all statistical analyses, confirm that the following items are present in the figure legend, table legend, main text, or Methods section.

n/a	Confirmed	
☐	☒	The exact sample size (*n*) for each experimental group/condition, given as a discrete number and unit of measurement
☐	☒	A statement on whether measurements were taken from distinct samples or whether the same sample was measured repeatedly
☐	☒	The statistical test(s) used AND whether they are one- or two-sided<br>*Only common tests should be described solely by name; describe more complex techniques in the Methods section.*
☐	☒	A description of all covariates tested
☐	☒	A description of any assumptions or corrections, such as tests of normality and adjustment for multiple comparisons
☐	☒	A full description of the statistical parameters including central tendency (e.g. means) or other basic estimates (e.g. regression coefficient) AND variation (e.g. standard deviation) or associated estimates of uncertainty (e.g. confidence intervals)
☐	☒	For null hypothesis testing, the test statistic (e.g. *F*, *t*, *r*) with confidence intervals, effect sizes, degrees of freedom and *P* value noted<br>*Give P values as exact values whenever suitable.*
☒	☐	For Bayesian analysis, information on the choice of priors and Markov chain Monte Carlo settings
☒	☐	For hierarchical and complex designs, identification of the appropriate level for tests and full reporting of outcomes
☒	☐	Estimates of effect sizes (e.g. Cohen's *d*, Pearson's *r*), indicating how they were calculated

Our web collection on statistics for biologists contains articles on many of the points above.

Software and code

Policy information about availability of computer code

Data collection	No software was used.
Data analysis	R version 3.6.2 (The R Foundation for Statistical Computing) or GraphPad Prism 8 were used to calculate statistic parameters and to draw graphs. Microsoft Excel for Microsoft 365 was used to draw graphs.<br>MD simulations were performed using Gromacs package 2018.1

For manuscripts utilizing custom algorithms or software that are central to the research but not yet described in published literature, software must be made available to editors and reviewers. We strongly encourage code deposition in a community repository (e.g. GitHub). See the Nature Portfolio guidelines for submitting code & software for further information.

Data

Policy information about availability of data

All manuscripts must include a data availability statement. This statement should provide the following information, where applicable:

- Accession codes, unique identifiers, or web links for publicly available datasets
- A description of any restrictions on data availability
- For clinical datasets or third party data, please ensure that the statement adheres to our policy

The mass spectrometry proteomics data of 5xFAD mice have been deposited to the ProteomeXchange Consortium via the PRIDE partner repository with the data set identifier PXD00129240.
Full size figures of Supplementary Figure 5b are available in the author’s Website (http://suppl.atgc.info/031/).
The mass spectrometry proteomics data of HMGB1 treated U2OS cells and human postmortem brains have been deposited to the ProteomeXchange Consortium

via the PRIDE partner repository with the data set identifier PXD028089.

Field-specific reporting

Please select the one below that is the best fit for your research. If you are not sure, read the appropriate sections before making your selection.

☒ Life sciences ☐ Behavioural & social sciences ☐ Ecological, evolutionary & environmental sciences

For a reference copy of the document with all sections, see nature.com/documents/nr-reporting-summary-flat.pdf

Life sciences study design

All studies must disclose on these points even when the disclosure is negative.

Sample size	Sample size is determined from previous reports from our and others' laboratories.
Data exclusions	There are no exclusion criteria for all analysis.
Replication	Experiments were independently repeated, the numbers of biological replicates are presented in the Figures.
Randomization	The selection of animals and the behavior analyses were performed by independent researchers.<br>Randomization (selection) of animals was simply dependent on chronological order of the birth date of animals.<br>The selection of images from immunohistochemistry/immunocytochemistry and the actual experiments of IHC/ICC were done by different researchers. In vitro live-cell imaging were done by different researchers.<br>Western blots are repeated until the necessary N was acquired.
Blinding	The information about group allocation or samples were opened to the data analyst or image acquisition researchers after finalizing results (m ake graphs etc).

Reporting for specific materials, systems and methods

We require information from authors about some types of materials, experimental systems and methods used in many studies. Here, indicate whether each material, system or method listed is relevant to your study. If you are not sure if a list item applies to your research, read the appropriate section before selecting a response.

Materials & experimental systems

n/a	Involved in the study	
☐	☒	Antibodies
☐	☒	Eukaryotic cell lines
☒	☐	Palaeontology and archaeology
☐	☒	Animals and other organisms
☒	☐	Human research participants
☒	☐	Clinical data
☒	☐	Dual use research of concern

Methods

n/a	Involved in the study	
☒	☐	ChIP-seq
☒	☐	Flow cytometry
☒	☐	MRI-based neuroimaging

Antibodies

Antibodies used	All antibodies used in the study are listed in the method.<br>Antibodies used for gel shift assay included Anti-Digoxigenin-AP antibody (1:10000, 3353591910, Roche, Basel, Switzerlan).<br>Antibodies used for immunohistochemistry included rabbit anti-phosphorylated Ser46-MARCKS (1:2000 [ordered from GL Biochem (Shanghai) Ltd., Shanghai, China]); mouse anti-amyloid beta (1:1000, clone 82E1, #10323, IBL, Gumma, Japan); rabbit anti-phospho-Ku70 (Ser77/Ser78) (1:1000[ordered from Cosmo Bio Co., Ltd., Tokyo, Japan]); mouse anti-Ku70 (1:250, E-5, Santa Cruz, Dallas, TX, USA); mouse anti-MAP2 (1:200, sc-32791, Santa Cruz Biotechnology, TX, USA); rabbit anti-MAP2 (1:2000, ab32454, Abcam, Abcam, Cambridge, UK); mouse anti-phospho-H2AX (gammaH2AX) (1:300, JBW301, Millipore, MA, USA); rabbit anti-53BP1 antibody (1:5000, NB100-304, Novus, CO, USA); rabbit anti-YAP (1:50, GTX129151, GeneTex, Irvine, CA, USA); rabbit anti-NFkB (1:100, #8242, Cell Signaling Technology, Danvers, MA, USA); mouse anti IL-6 (1:50, ab208113, Abcam, Cambridge, UK); rabbit anti-CCL2/MCP1 (1:100, NBP1-07035, Novus, CO, USA); rabbit anti-pSer232-RIP3 (1:200, ab195117, Abcam, Cambridge, UK); rabbit anti-pSer345-MLKL (1:500, ab196436, Abcam, Cambridge, UK); rabbit anti-Lamin B1 antibody (1:5000, ab16048, Abcam, Cambridge, UK); rabbit anti-cleaved Caspase1 (1:100, #89332, Cell Signaling Technology, Danvers, MA, USA); rabbit anti-cleaved Caspase9 (1:200, #9509, Cell Signaling Technology, Danvers, MA, USA); goat anti-Iba1 (1:500, #011-27991, Wako, Osaka, Japan); rabbit anti-Iba1 (1:100, #019-19741, Wako, Osaka, Japan); mouse anti-GFAP Cy3-conjugated (1:5000, #C9205, Sigma-Aldrich, St.Louis, MO, USA); mouse anti-NeuN (1:1000, ab104224, Abcam, Cambridge, UK); rabbit anti-PSD95 (1:100, D74D3, Cell Signaling Technology, Danvers, MA, USA); and rabbit anti-phospho-Tau (phospho S214) (1:2000 , ab170892, Abcam, Cambridge, UK) antibodies. The reaction products were visualized by Alexa Fluor 488-, 568- and 647- conjugated secondary antibody (1:1000, Molecular Probes, MA, USA).<br>Antibodies used for westernblot included rabbit anti-phospho-Ku70 (Ser77/Ser78) (1:100000[ordered from Cosmo Bio Co., Ltd.,

Tokyo, Japan]); rabbit anti-GST(z-5) (1:5000, sc-459, Santa Cruz Biotechnology, Dallas, TX, USA); mouse anti-amyloid beta(1:5000, clone 82E1, #10323, IBL, Gumma, Japan); mouse anti-Histone H4 (1:5000, ab31830, Abcam, Cambridge, UK); mouse anti-□-actin (1:1000, sc-8334, Santa Cruz Biotechnology, TX, USA); rabbit anti-phosphorylated Ser46-MARCKS (1:100,000, GL Biochem Ltd., Shanghai, China) mouse anti-MARCKS (1:1000, sc-100777, Santa Cruz Biotechnology, TX, USA); mouse anti-amyloid beta(1:3000, clone 82E1, IBL, Gumma, Japan); mouse anti-phospho-H2AX (gammaH2AX) (1:3000, JBW301, Millipore, Burlington, MA, USA); rabbit anti-53BP1 (1:15,000, NB100-304, Novus, CO, USA); rabbit anti-phospho-p44/42 MAPK (Erk1/2) (phospho Thr202/Tyr204) (1:30000, 4370, Cell Signaling Technology, Danvers, MA, USA); rabbit anti-p44/42 MAPK (Erk1/2) (1:5000, 9102, Cell Signaling, Danvers, MA, USA); rabbit anti-phospho-CDK1 (phospho T14)(1:5000, ab58509, Abcam, Cambridge, UK); mouse anti-CDK1(1:5000, ab18, Abcam); rabbit anti-Ku70(phosphor Ser5) (1:3000, PA5-40427, Thermo Fisher Scientific, Waltham, MA, USA); goat anti-Ku70 (M-19) (1:3000, sc-1487, Santa Cruz Biotechnology, Dallas, TX, USA); mouse anti-phospho-ATM (phospho S1981) (1:10000, 200-301-400, Rockland, PA, USA); rabbit anti-ATM (1:3000, PC85-100UG, EMD Chemicals, MA, USA); rabbit anti-phospho-ATR (phospho Ser428) (1:5000, 2853, Cell Signaling, Danvers, MA, USA); mouse anti-ATR (C-1) (1:3000, sc-515173, Santa Cruz Biotechnology, Dallas, TX, USA); rabbit anti-phospho-Tau (phospho Ser214)(1:15000, ab170892, Abcam, Cambridge, UK); rabbit anti-phospho-PKC□ (phospho Thr638) (1:20,000 ab32502, Abcam, Cambridge, UK); rabbit anti-phospho-PKCbetaI (phospho Thr641) (1:1000, sc-101776, Santa Cruz Biotechnology, Dallas, TX, USA); goat anti-phospho-PKCbetaII/delta (phospho Thr660) (1:10000, sc-11760, Santa Cruz Biotechnology, Dallas, TX, USA); rabbit anti-phospho-MEK1/2 (phospho Ser217/221) (1:50000, 9121, Cell Signaling Technology, Dallas, TX, USA); rabbit-MEK1 (1:3000, ab32091, Abcam, Dallas, TX, USA); rabbit anti-pan-PKC (1:1500, GTX52352, GeneTex, Irvine, CA, USA); rabbit anti-Ku70(acetyl K331) antibody (1:5000, ab190626, Abcam, Cambridge, UK); mouse-anti SIRT1(1:2000, #8469S, Cell Signaling Technology, Danvers, MA, USA); rabbit anti-14-3-3(1:1000, #14503-1-AP, Protein Tech,); rabbit anti-Ku80(1:1000, sc-9034, Santa Cruz Biotechnology, Dallas, TX, USA); rabbit anti-DNA-PKcs(1:5000, ab32566, Abcam, Cambridge, UK); rabbit anti-TDP43(1:1000, ab109535, Abcam, Cambridge, UK); VCP(1:1000, #612182, BD bioscience , San Jose, CA, USA); rabbit anti-EGFP(1:5000, sc-8334, Santa Cruz Biotechnology, Dallas, TX, USA); mouse anti-Tau (1:5000, ab80579, abcam, Cambridge, UK); HRP-linked anti-rabbit IgG (1:3000, NA934, GE Healthcare, Buckinghamshire, United Kingdom); HRP-linked anti-mouse IgG, 1:3000 (NA931, GE Healthcare, Buckinghamshire, United Kingdom); and donkey anti-goat IgG-HRP (1:3000, sc-2020, Santa Cruz Biotechnology, Dallas, TX, USA) antibodies.
Antibodies used for immunocytochemistry included rabbit-anti-AC3 (1:500, sc-588, Santa Cruz Biotechnology, Dallas, TX, USA), mouse-anti-MAP2 (1:250, sc-32791, Santa Cruz Biotechnology, Dallas, TX, USA), Alexa Fluor 488–conjugated anti-rabbit IgG (1:1000, A21206, Molecular Probes, Eugene, OR, USA), and Alexa Fluor 647–conjugated anti-mouse IgG (1:1000, A31571, Molecular Probes, Eugene, OR, USA) antibodies.

Validation

Information of validation of commercially available antibodies are provided on the manufacturer's websites. Rabbit anti-pSer46-MARCKS antibody was originally prepared and was validated in our previous publication (ref#38, Fujita et al., 2016, Sci Rep, doi:10.1038/srep31895).

Eukaryotic cell lines

Policy information about cell lines

Cell line source(s)

U2OS cells were kindly gifted from Dr. Yoshio Miki (Tokyo Medical and Dental University).

Authentication

None of cell lines used were authenticated.

Mycoplasma contamination

All cell lines were negative for mycoplasma contamination.

Commonly misidentified lines
(See ICLAC register)

We did not use any misidentified cell lines.

Animals and other organisms

Policy information about studies involving animals; ARRIVE guidelines recommended for reporting animal research

Laboratory animals

We wrote the species, strain, and sex in the method, and age of animals were indicated in the figure and figure legends ("figure legends").

Wild animals

The study did not involve any wild animals.

Field-collected samples

The study did not involve any samples collected from the field.

Ethics oversight

We wrote the statement that our experiments follow ethical regulations in the method ("Ethics for animal experiments" and "Ethics for human experiments").

Note that full information on the approval of the study protocol must also be provided in the manuscript.
